# Supplementary material for: HOOK2 downregulation compromises the tumorigenic and stemness properties of ovarian cancer cells by increasing endoplasmic reticulum stress
Source: Cell Death Dis. 2026 Apr 24;17(1):546. doi: 10.1038/s41419-026-08763-5 (PMC13243538; doi:10.1038/s41419-026-08763-5)
Supplement: Supplementary file 1 — Supplementary material [file 41419_2026_8763_MOESM1_ESM.pdf]

SUPPLEMENTARY DATA

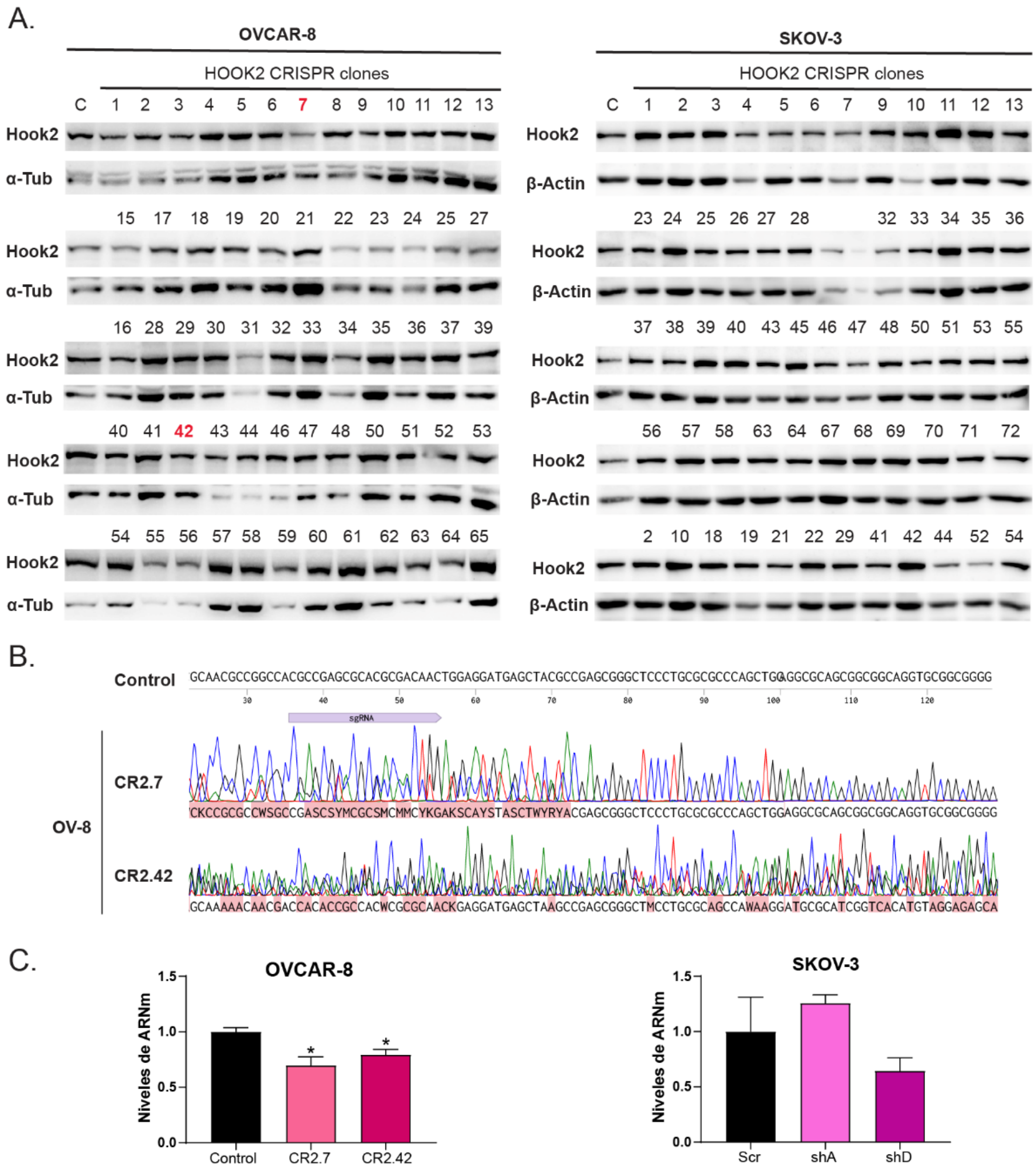

Supplementary Figure S1. Validation of the HOOK2 reduction models generated in ovarian cancer cell lines OVCAR-8 and SKOV-3. (A) WB analysis of HOOK2 expression in HOOK2 CRISPR clones within OVCAR-8 and SKOV-3 cell lines. Clones selected for use in the OVCAR-8 line are highlighted in red. **(B)** DNA sequencing of the sgRNA region in the selected CRISPR clones in the OVCAR-8 line, compared to the original sequence. **(C)** q-RT-PCR of HOOK2 expression in selected CRISPR clones in the OVCAR-8 line and selected shRNAs in the SKOV-3 line. The mean of 3 independent experiments ± SEM is presented. Statistical analysis was performed with Student's t test (*p < 0.05; **p < 0.01; ***p < 0.001). The lack of an asterisk indicates that the data do not reach statistical significance.

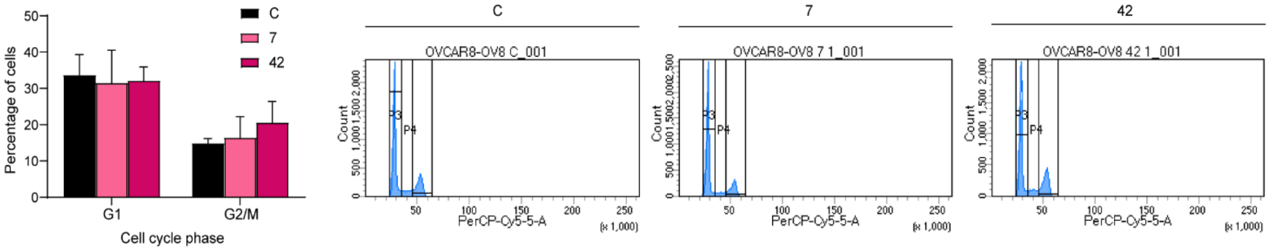

Supplementary Figure S2. Cell cycle is not altered upon HOOK2 depletion. Quantification by flow cytometry of cells in G1 and G2/M phases in OVCAR-8 ovarian cancer cells after HOOK2 knockdown. The mean of 3 independent experiments ± SEM is presented. Statistical analysis was performed with Student's t test (*$p < 0.05$; **$p < 0.01$; ***$p < 0.001$). The lack of an asterisk indicates that the data do not reach statistical significance.

A.

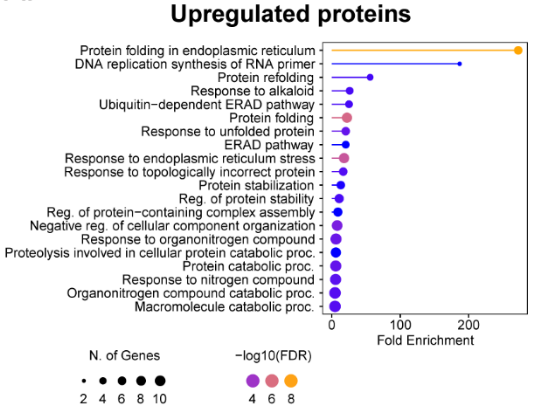

B.

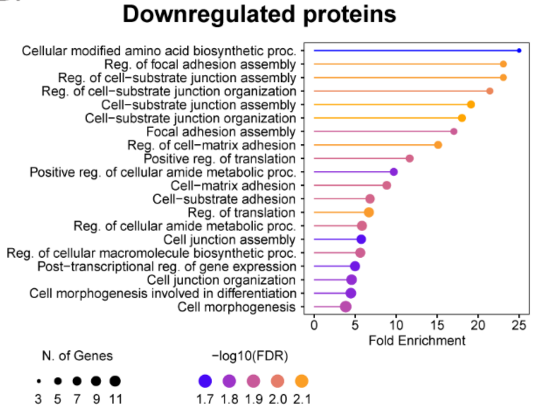

C. Upregulated proteins

Process	Terms	Associated Proteins Found
Positive regulation of mononuclear cell migration	Positive regulation of mononuclear cell migration (GO:0071677)	CALR, LGALS3, SERPINE1
Regulation of response to endoplasmic reticulum stress	Negative regulation of response to endoplasmic reticulum stress (GO:1903573)	HSPA1A, HSPA5, HYOU1, USP14
	Regulation of response to endoplasmic reticulum stress (GO:1905897)	HSPA1A, HSPA5, HYOU1, MANF, USP14
Protein folding in the endoplasmic reticulum	Protein folding in endoplasmic reticulum (GO:0034975)	CALR, DNAJC3, HSP90B1, HSPA5, PDIA3
	Ubiquitin-dependent ERAD pathway (GO:0030433)	CALR, HSP90B1, HSPA5, USP14
Protein folding chaperone	'De novo' protein folding (GO:0006458)	HSPA1A, HSPA1B, HSPA5, HSPA8
	Protein refolding (GO:0042026)	HSP90AA1, HSPA1A, HSPA1B, HSPA5, HSPA8
	Protein folding chaperone (GO:0044183)	CALR, HSP90AA1, HSPA1A, HSPA1B, HSPA5, HSPA8
	Chaperone-mediated protein folding (GO:0061077)	HSPA1A, HSPA1B, HSPA5, HSPA8, PDIA4
	Response to topologically incorrect protein (GO:0035966)	DNAJC3, HSP90AA1, HSPA1A, HSPA1B, HSPA5, HSPA8, MANF
	'De novo' posttranslational protein folding (GO:0051084)	HSPA1A, HSPA1B, HSPA5, HSPA8
	Response to unfolded protein (GO:0006986)	DNAJC3, HSP90AA1, HSPA1A, HSPA1B, HSPA5, HSPA8, MANF
	Interleukin-8 production (GO:0032637)	HSPA1A, HSPA1B, RAB1A, SERPINE1
	Cellular response to heat (GO:0034605)	HSP90AA1, HSPA1A, HSPA1B
	Chaperone cofactor-dependent protein refolding (GO:0051085)	HSPA1A, HSPA1B, HSPA5, HSPA8
	Regulation of interleukin-8 production (GO:0032677)	HSPA1A, HSPA1B, RAB1A, SERPINE1
	Positive regulation of interleukin-8 production (GO:0032757)	HSPA1A, HSPA1B, RAB1A, SERPINE1

D. Downregulated proteins

Process	Term	Associated Genes Found
Peptidyl-proline modification	Peptidyl-proline modification (GO:0018208)	FKBP4, FKBP5, P4HA1
Cellular modified amino acid biosynthetic process	Cellular modified amino acid biosynthetic process (GO:0042398)	ALDH7A1, CKB, GAMT
Cell substrate junction assembly	Cell-substrate junction organization (GO:0150115)	FERMT2, LAMC1, SLK, THBS1, VCL
	Regulation of cell-substrate junction organization (GO:0150116)	FERMT2, SLK, THBS1, VCL
	Cell-substrate junction assembly (GO:0007044)	FERMT2, LAMC1, SLK, THBS1, VCL
	Focal adhesion assembly (GO:0048041)	FERMT2, SLK, THBS1, VCL
	Regulation of cell-substrate junction assembly (GO:0090109)	FERMT2, SLK, THBS1, VCL
	Regulation of focal adhesion assembly (GO:0051893)	FERMT2, SLK, THBS1, VCL

E.

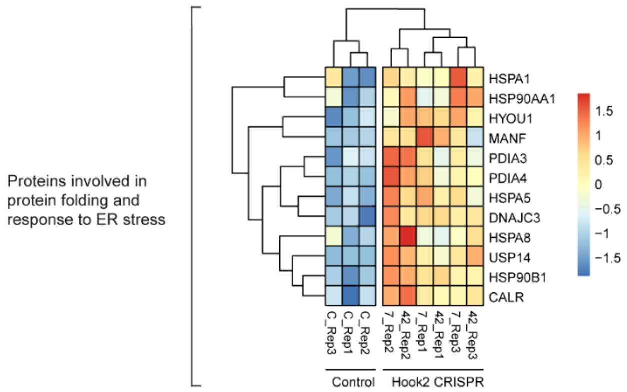

Supplementary Figure S3. Ontology term analysis of proteins altered by the downregulation of HOOK2 levels. Ontology term analysis of **(A)** upregulated and **(B)** downregulated proteins upon HOOK2 reduction performed in the ShinyGO 0.80 web resource. Breakdown of proteins **(C)** upregulated and **(D)** downregulated upon HOOK2 reduction implicated in the processes depicted in Figure 2B and C. **(E)** Heatmap of relative normalized spectral counts of proteins involved in protein folding and response to ER stress in cells with downregulated HOOK2.

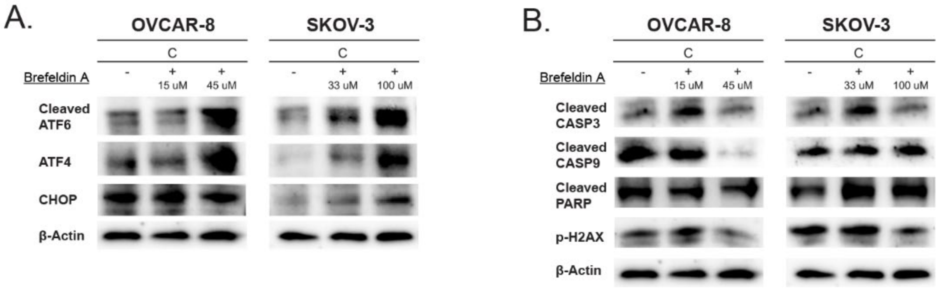

Supplementary Figure S4. **Validation of brefeldin A treatment.** Protein levels of **(A)** UPR-associated proteins and **(B)** apoptosis-associated proteins in ovarian cancer cells treated with different doses of the ER stress inducer brefeldin A.

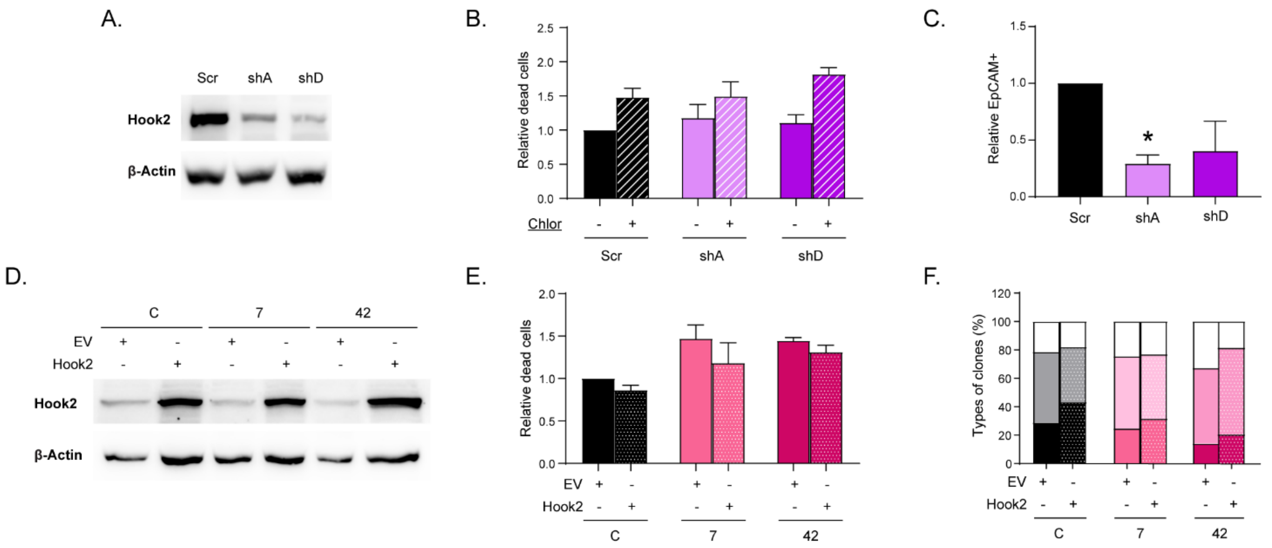

Supplementary Figure S5. Validation of HOOK2 downregulation specificity using an alternative knockdown strategy and rescuing HOOK2 levels in the OVCAR-8 cell line. A) Validation of decreased HOOK2 levels in ovarian cancer cell line OVCAR-8 infected with shRNAs against HOOK2. **(B)** Quantification by flow cytometry of the relative number of dead cells when HOOK2 is downregulated. The mean of 3 independent experiments ± SEM is presented. Statistical analysis was performed with Student's t test (*p < 0.05; **p < 0.01; ***p < 0.001). The lack of an asterisk indicates that the data do not reach statistical significance. **(C)** Quantification of the relative number of cells expressing the EpCAM marker in cells with reduced HOOK2. The mean of 3 independent experiments ± SEM is presented. Statistical analysis was performed with Student's t test (*p < 0.05; **p < 0.01; ***p < 0.001). The lack of an asterisk indicates that the data do not reach statistical significance. **(D)** Validation of increased HOOK2 levels in cell line OVCAR-8 transfected with a plasmid constitutively expressing HOOK2. **(E)** Quantification by flow cytometry of the relative number of dead cells following HOOK2 upregulation in HOOK2 CRISPR cells. The mean of 3 independent experiments ± SEM is presented. Statistical analysis was performed with Student's t test (*p < 0.05; **p < 0.01; ***p < 0.001). The lack of an asterisk indicates that the data do not reach statistical significance. **(F)** Assessment of the percentage of holoclones, meroclones, and paraclones in a clonability assay of HOOK2 CRISPR cells with HOOK2 overexpression. The mean of 1 independent experiment is presented.

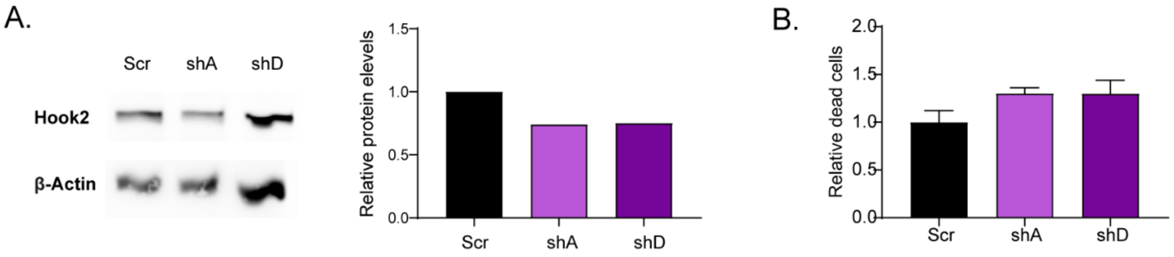

Supplementary Figure S6. Impact of HOOK2 knockdown on non-tumoral cell survival. (A) HOOK2 knockdown using shRNAs in the non-tumoral ovarian cell line FT282. **(B)** Quantification by flow cytometry of the relative percentage of dead cells when HOOK2 is downregulated. The mean of 2 independent experiments ± SEM is presented. Statistical analysis was performed with Student's t test (*p < 0.05; **p < 0.01; ***p < 0.001). The lack of an asterisk indicates that the data do not reach statistical significance.

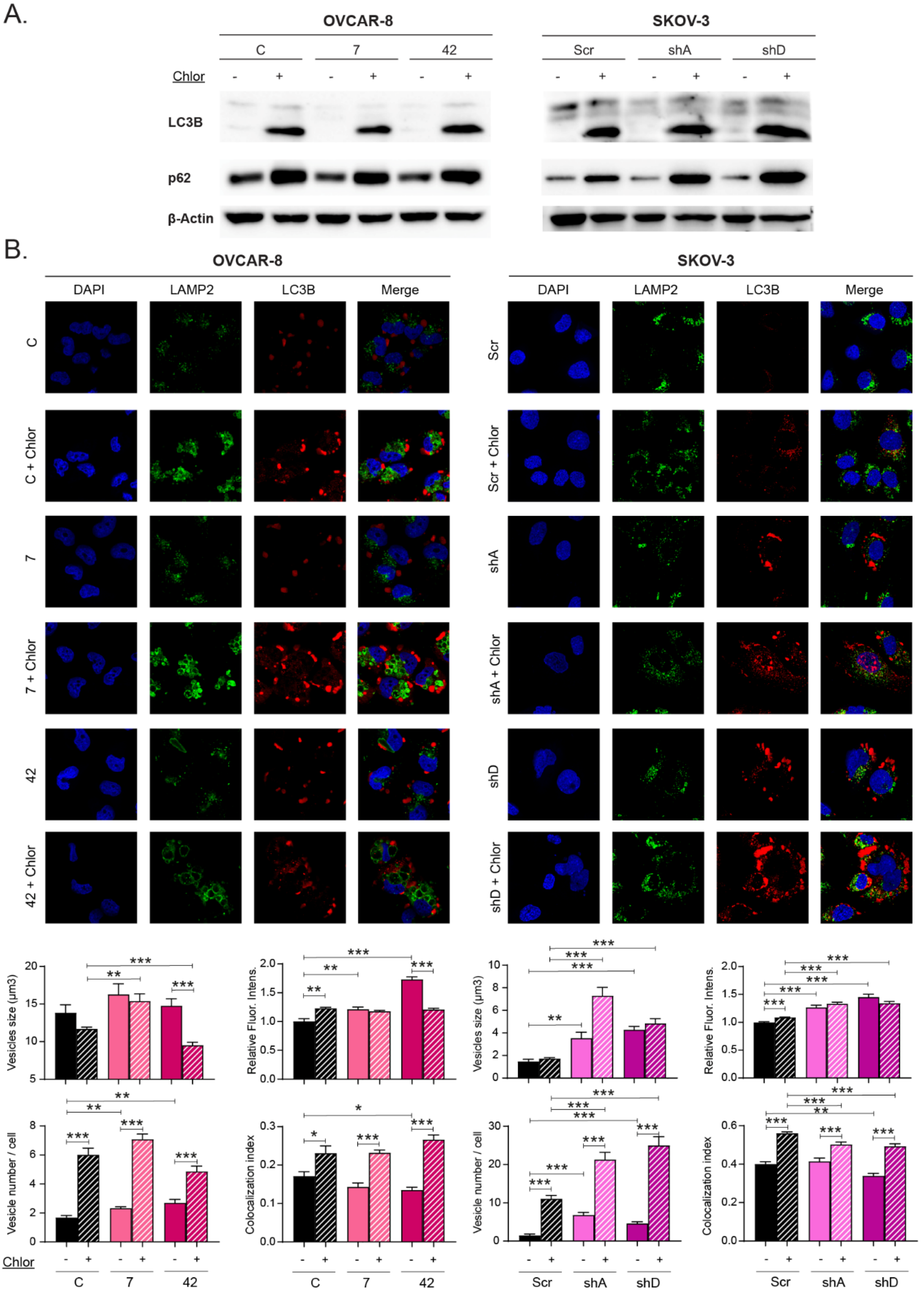

Supplementary Figure S7. Validation of chloroquine treatment. (A) Protein levels of autophagy-associated proteins in HOOK2-downregulated cells treated with chloroquine. **(B)** Immunofluorescence staining of LAMP2 (in green) and LC3B (in red) proteins in HOOK2-downregulated cells treated with chloroquine. DAPI (in blue) was utilized for nuclear staining,

and the merge of the three markers is presented. Autophagosome size, number, relative fluorescence intensity, and the colocalization index of autophagosomes-lysosomes were quantified using ImageJ software. Analysis involved a minimum of 100 cells for each condition. Statistical analysis was performed with Student's t test (*$p < 0.05$; **$p < 0.01$; ***$p < 0.001$). The lack of an asterisk indicates that the data do not reach statistical significance.

Supplementary Figure S1. Validation of the HOOK2 reduction models generated in ovarian cancer cell lines OVCAR-8 and SKOV-3. (A) WB analysis of HOOK2 expression in HOOK2 CRISPR clones within OVCAR-8 and SKOV-3 cell lines. Clones selected for use in the OVCAR-8 line are highlighted in red. **(B)** DNA sequencing of the sgRNA region in the selected CRISPR clones in the OVCAR-8 line, compared to the original sequence. **(C)** q-RT-PCR of HOOK2 expression in selected CRISPR clones in the OVCAR-8 line and selected shRNAs in the SKOV-3 line. The mean of 3 independent experiments ± SEM is presented. Statistical analysis was performed with Student's t test (*p < 0.05; **p < 0.01; ***p < 0.001). The lack of an asterisk indicates that the data do not reach statistical significance.

Supplementary Figure S2. Cell cycle is not altered upon HOOK2 depletion. Quantification by flow cytometry of cells in G1 and G2/M phases in OVCAR-8 ovarian cancer cells after HOOK2 knockdown. The mean of 3 independent experiments ± SEM is presented. Statistical analysis was performed with Student's t test (*p < 0.05; **p < 0.01; ***p < 0.001). The lack of an asterisk indicates that the data do not reach statistical significance.

Supplementary Figure S3. Ontology term analysis of proteins altered by the downregulation of HOOK2 levels. Ontology term analysis of **(A)** upregulated and **(B)** downregulated proteins upon HOOK2 reduction performed in the ShinyGO 0.80 web resource. Breakdown of proteins **(C)** upregulated and **(D)** downregulated upon HOOK2 reduction implicated in the processes depicted in Figure 2B and C. **(E)** Heatmap of relative normalized spectral counts of proteins involved in protein folding and response to ER stress in cells with downregulated HOOK2.

Supplementary Figure S4. **Validation of brefeldin A treatment.** Protein levels of **(A)** UPR-associated proteins and **(B)** apoptosis-associated proteins in ovarian cancer cells treated with different doses of the ER stress inducer brefeldin A.

Supplementary Figure S5. Validation of HOOK2 downregulation specificity using an alternative knockdown strategy and rescuing HOOK2 levels in the OVCAR-8 cell line. A) Validation of decreased HOOK2 levels in ovarian cancer cell line OVCAR-8 infected with shRNAs against HOOK2. **(B)** Quantification by flow cytometry of the relative number of dead cells when HOOK2 is downregulated. The mean of 3 independent experiments ± SEM is presented. Statistical analysis was performed with Student's t test (*p < 0.05; **p < 0.01; ***p < 0.001). The lack of an asterisk indicates that the data do not reach statistical significance. **(C)** Quantification of the relative number of cells expressing the EpCAM marker in cells with reduced HOOK2. The mean of 3 independent experiments ± SEM is presented. Statistical analysis was performed with Student's t test (*p < 0.05; **p < 0.01; ***p < 0.001). The lack of an asterisk indicates that the data do not reach statistical significance. **(D)** Validation of increased HOOK2 levels in cell line OVCAR-8 transfected with a plasmid constitutively expressing HOOK2. **(E)** Quantification by flow cytometry of the relative number of dead cells following HOOK2 upregulation in HOOK2 CRISPR cells. The mean of 3 independent experiments ± SEM is presented. Statistical analysis was performed with Student's t test (*p < 0.05; **p < 0.01; ***p < 0.001). The lack of an asterisk indicates that the data do not reach statistical significance. **(F)** Assessment of the percentage of holoclones, meroclones, and paraclones in a clonability assay of HOOK2 CRISPR cells with HOOK2 overexpression. The mean of 1 independent experiment is presented.

Supplementary Figure S6. Impact of HOOK2 knockdown on non-tumoral cell survival. (A) HOOK2 knockdown using shRNAs in the non-tumoral ovarian cell line FT282. **(B)** Quantification by flow cytometry of the relative percentage of dead cells when HOOK2 is downregulated. The mean of 2 independent experiments ± SEM is presented. Statistical analysis was performed with Student's t test (*p < 0.05; **p < 0.01; ***p < 0.001). The lack of an asterisk indicates that the data do not

reach statistical significance.

Supplementary Figure S7. Validation of chloroquine treatment. (A) Protein levels of autophagy-associated proteins in HOOK2-downregulated cells treated with chloroquine. **(B)** Immunofluorescence staining of LAMP2 (in green) and LC3B (in red) proteins in HOOK2-downregulated cells treated with chloroquine. DAPI (in blue) was utilized for nuclear staining, and the merge of the three markers is presented. Autophagosome size, number, relative fluorescence intensity, and the colocalization index of autophagosomes-lysosomes were quantified using ImageJ software. Analysis involved a minimum of 100 cells for each condition. Statistical analysis was performed with Student's t test (*p < 0.05; **p < 0.01; ***p < 0.001). The lack of an asterisk indicates that the data do not reach statistical significance.

UNCROPPED WESTERN BLOTS
