# Supplementary material for: HOOK2 downregulation compromises the tumorigenic and stemness properties of ovarian cancer cells by increasing endoplasmic reticulum stress
Source: Cell Death Dis. 2026 Apr 24;17(1):546. doi: 10.1038/s41419-026-08763-5 (PMC13243538; doi:10.1038/s41419-026-08763-5)
Supplement: Supplementary file 2 — Original data [file 41419_2026_8763_MOESM2_ESM.pdf]

Figure 1C

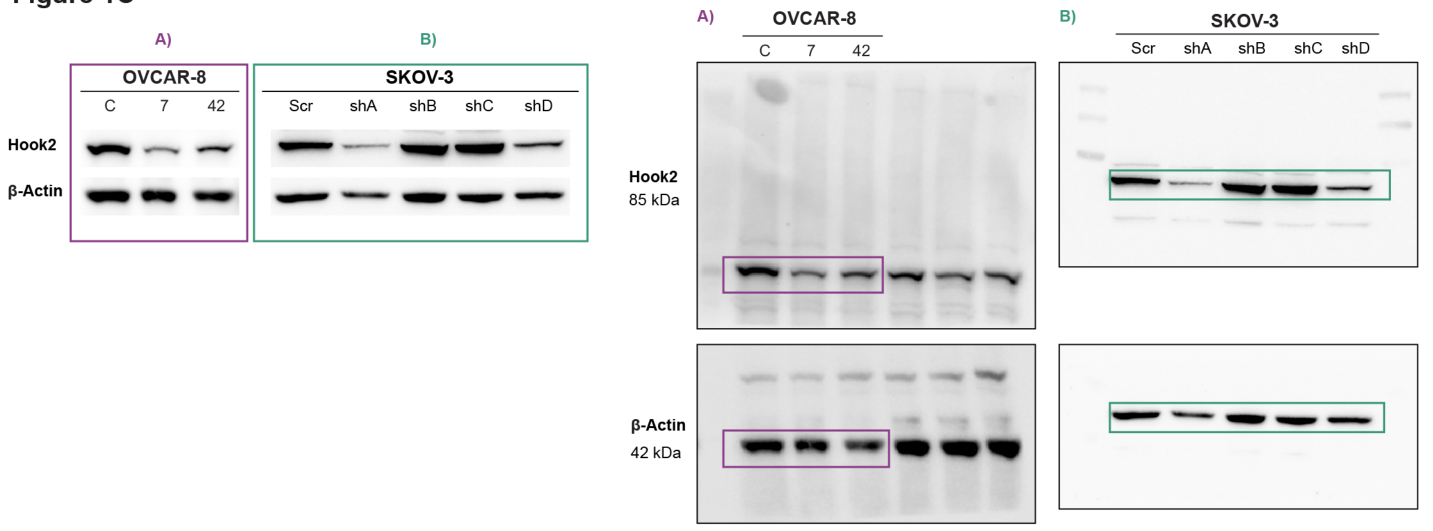

Figure 3A

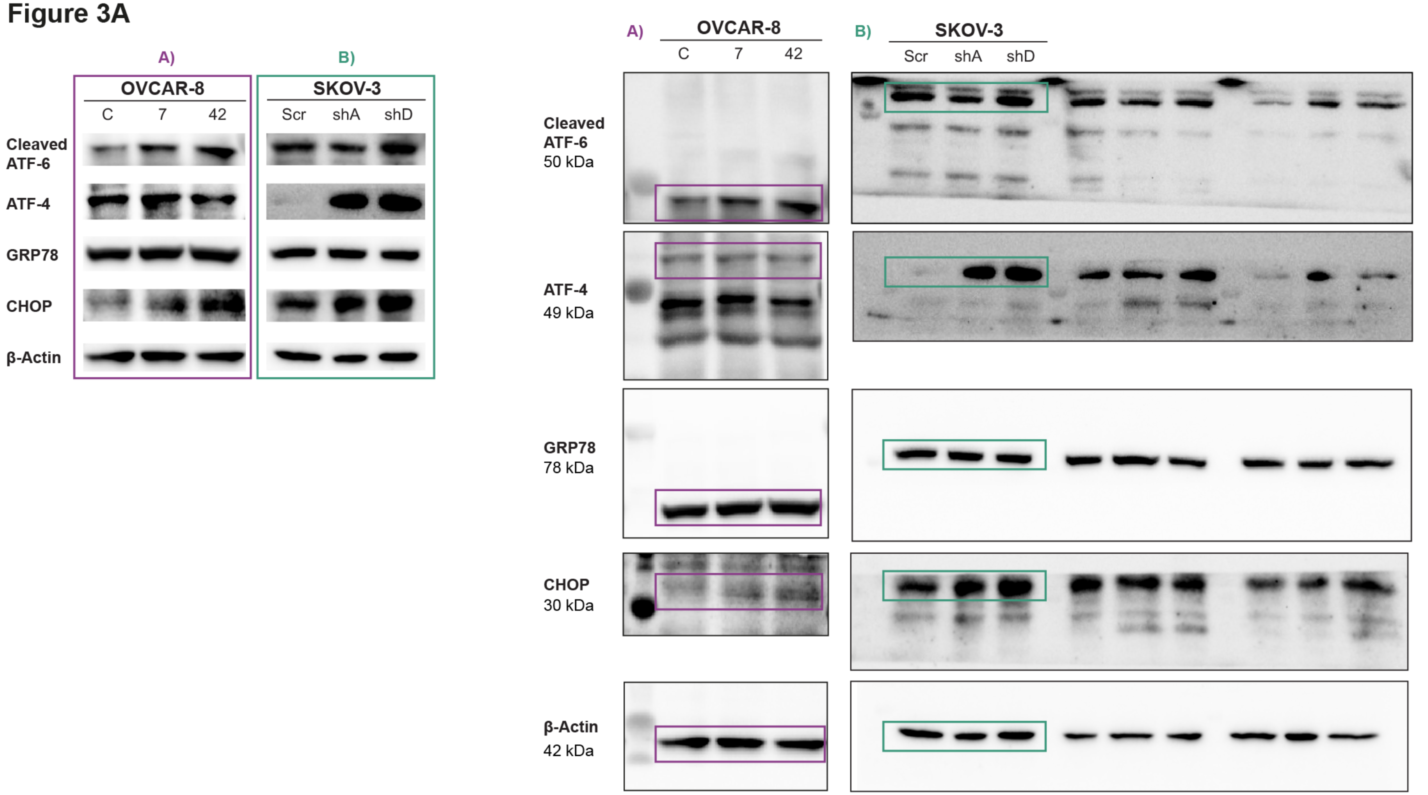

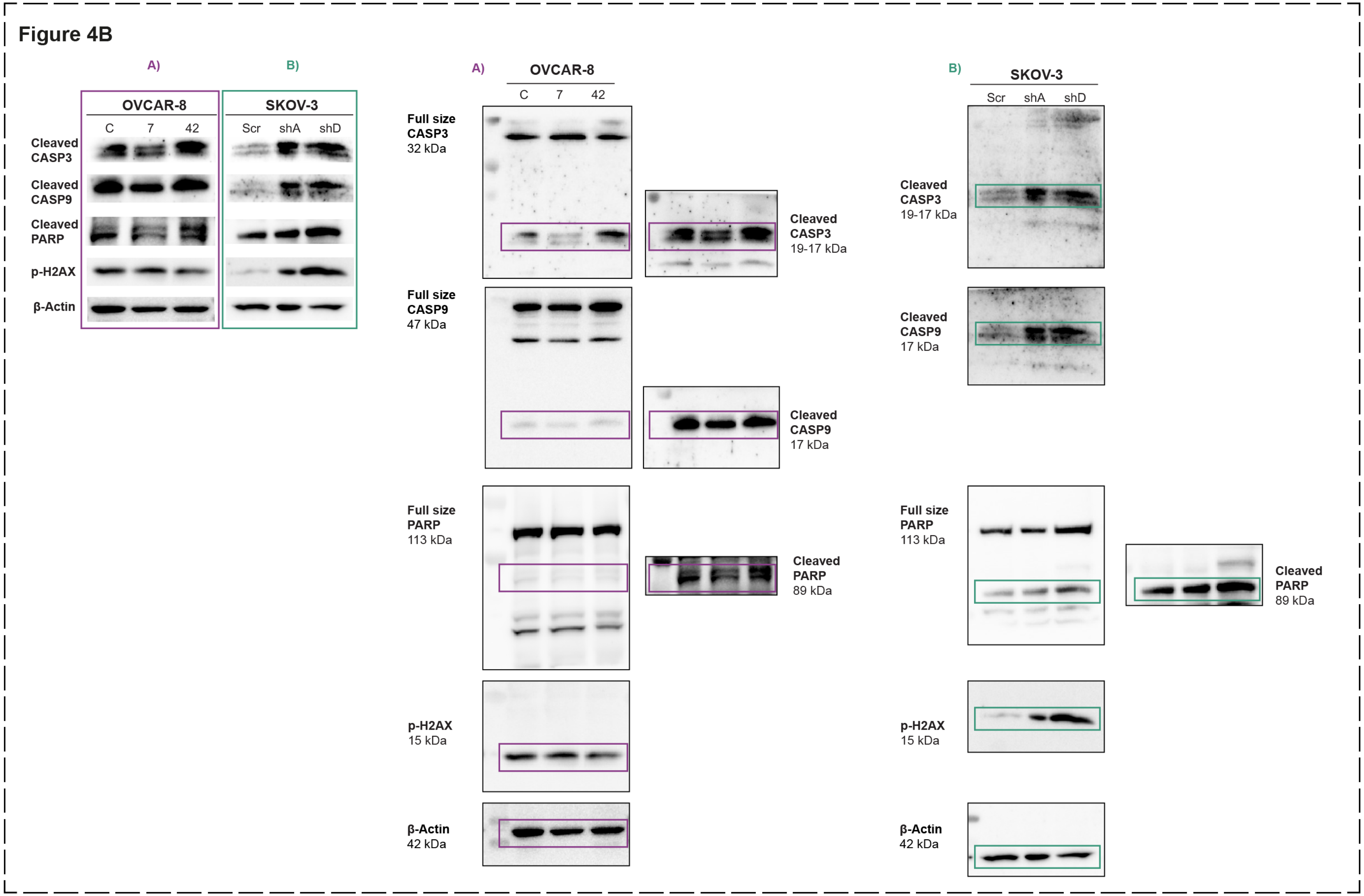

Figure 4B
A)
B)
OVCAR-8
SKOV-3
C
7
42
Scr
shA
shD
Cleaved CASP3
Cleaved CASP9
Cleaved PARP
p-H2AX
β-Actin
A)
OVCAR-8
C
7
42
Full size CASP3 32 kDa
Cleaved CASP3 19-17 kDa
Full size CASP9 47 kDa
Cleaved CASP9 17 kDa
Full size PARP 113 kDa
Cleaved PARP 89 kDa
p-H2AX 15 kDa
β-Actin 42 kDa
B)
SKOV-3
Scr
shA
shD
Cleaved CASP3 19-17 kDa
Cleaved CASP9 17 kDa
Full size PARP 113 kDa
Cleaved PARP 89 kDa
p-H2AX 15 kDa
β-Actin 42 kDa

Figure 4D

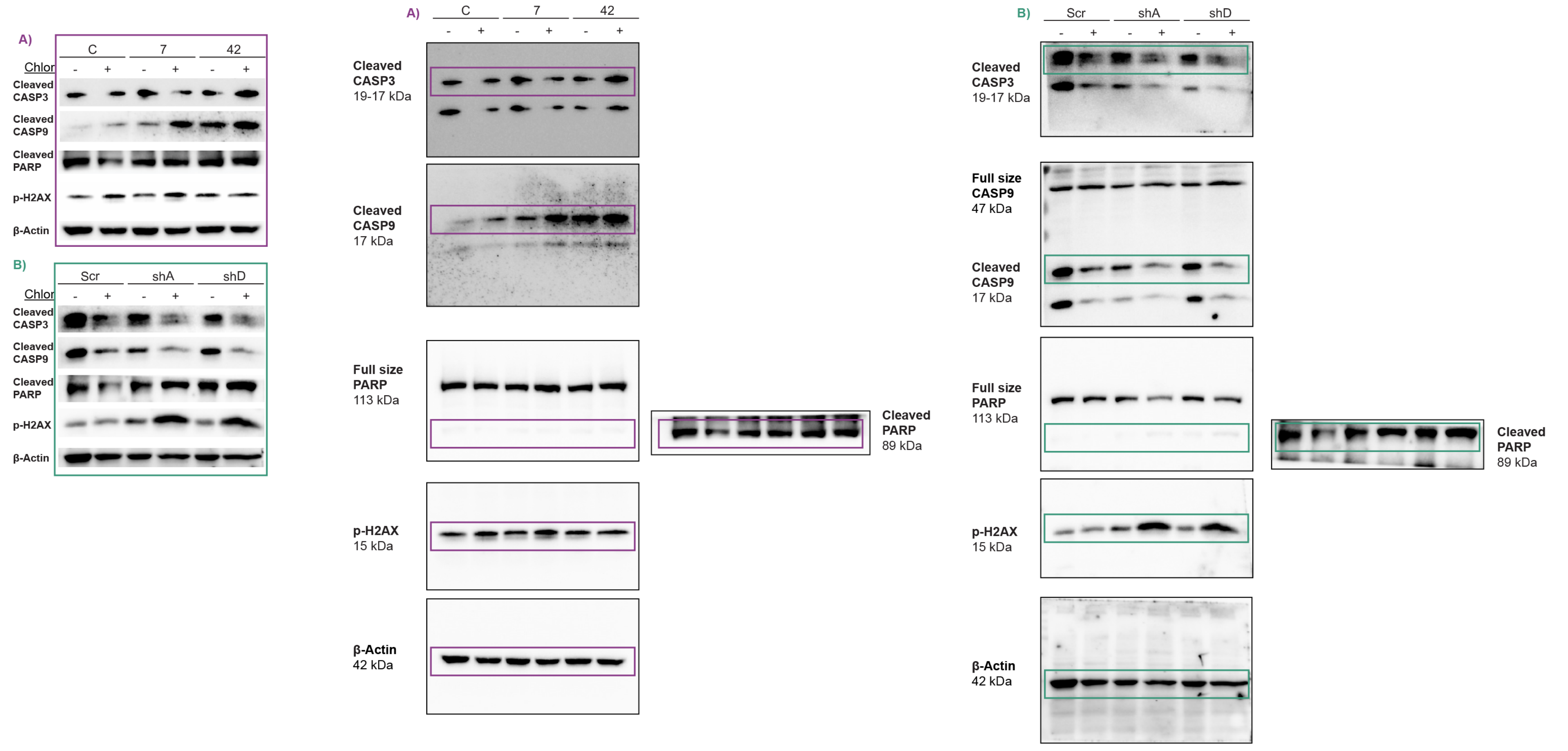

A)
C
7
42
Chlor
- + - + - +
Cleaved CASP3
Cleaved CASP9
Cleaved PARP
p-H2AX
β-Actin
B)
Scr
shA
shD
Chlor
- + - + - +
Cleaved CASP3
Cleaved CASP9
Cleaved PARP
p-H2AX
β-Actin
A)
C
7
42
- + - + - +
Cleaved CASP3 19-17 kDa
Cleaved CASP9 17 kDa
Full size PARP 113 kDa
Cleaved PARP 89 kDa
p-H2AX 15 kDa
β-Actin 42 kDa
B)
Scr
shA
shD
- + - + - +
Cleaved CASP3 19-17 kDa
Full size CASP9 47 kDa
Cleaved CASP9 17 kDa
Full size PARP 113 kDa
Cleaved PARP 89 kDa
p-H2AX 15 kDa
β-Actin 42 kDa

Supplementary Figure 1A

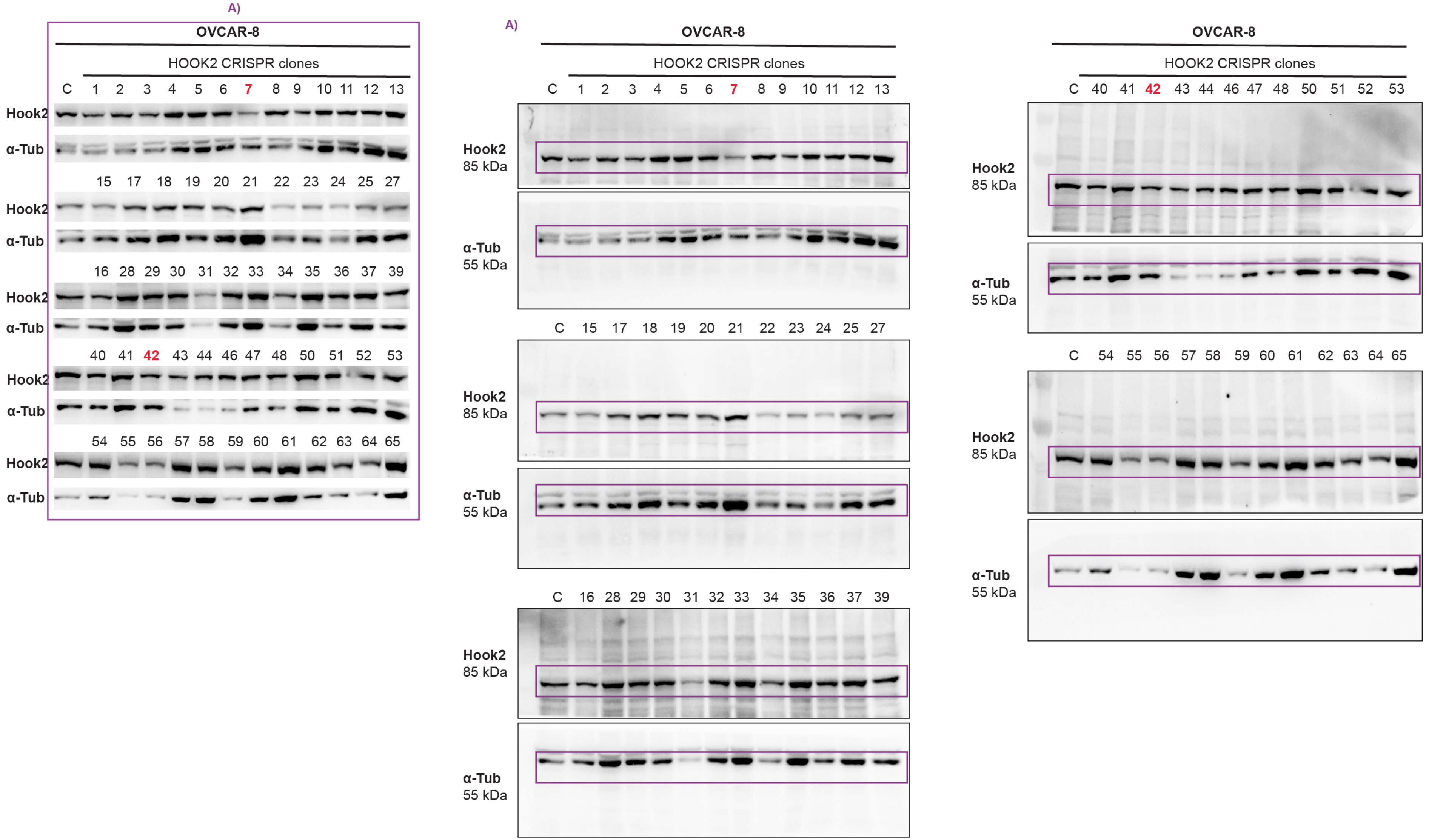

Supplementary Figure 1A

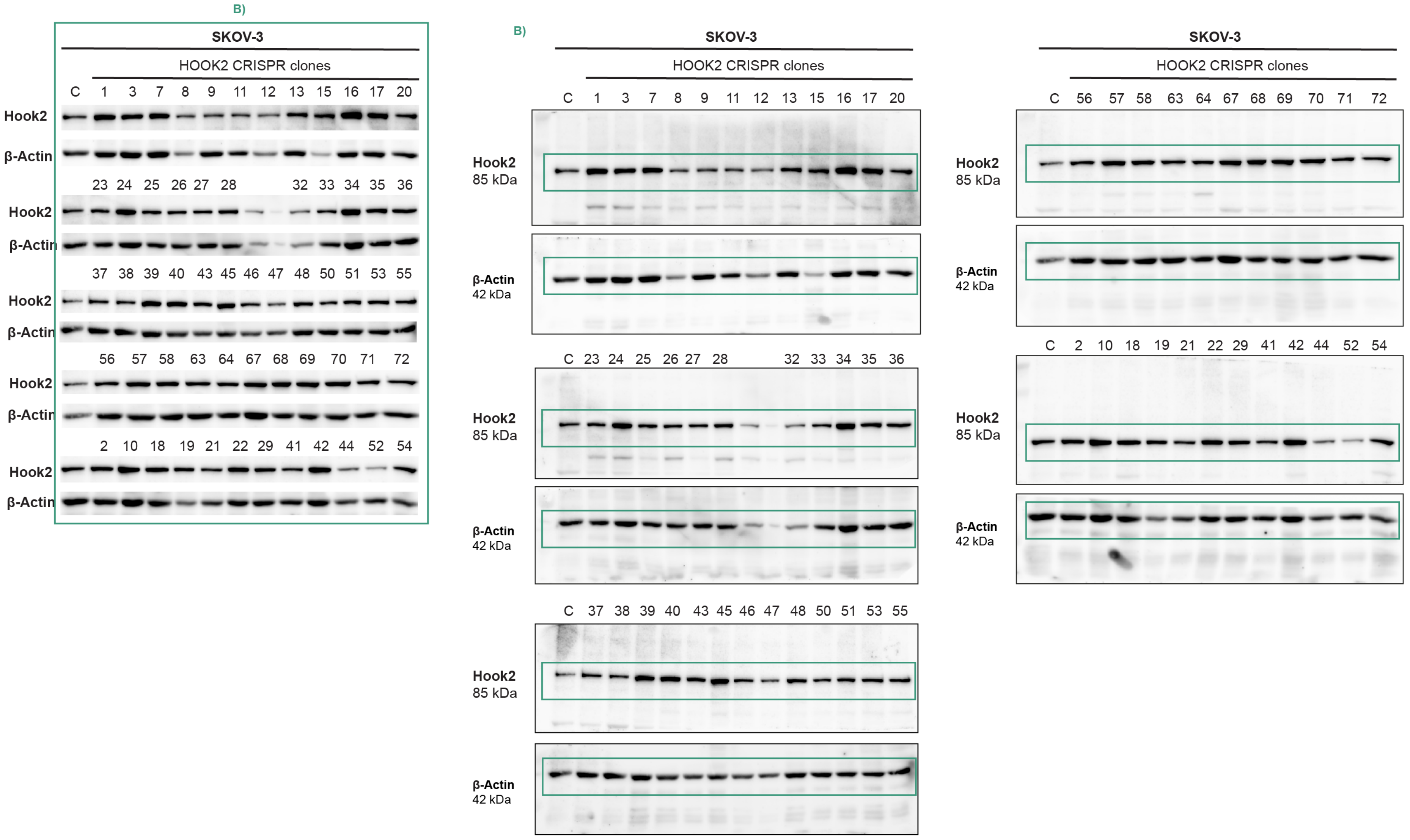

Supplementary Figure 4A

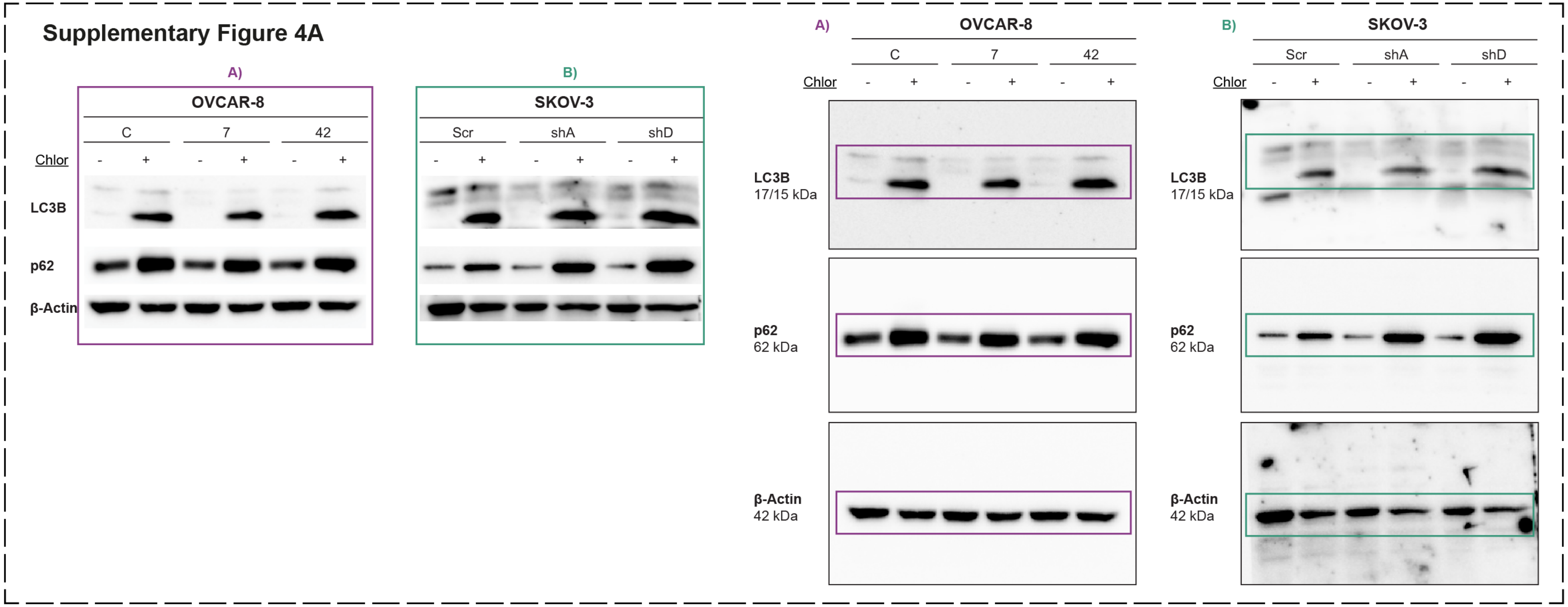

Supplementary Figure 5A

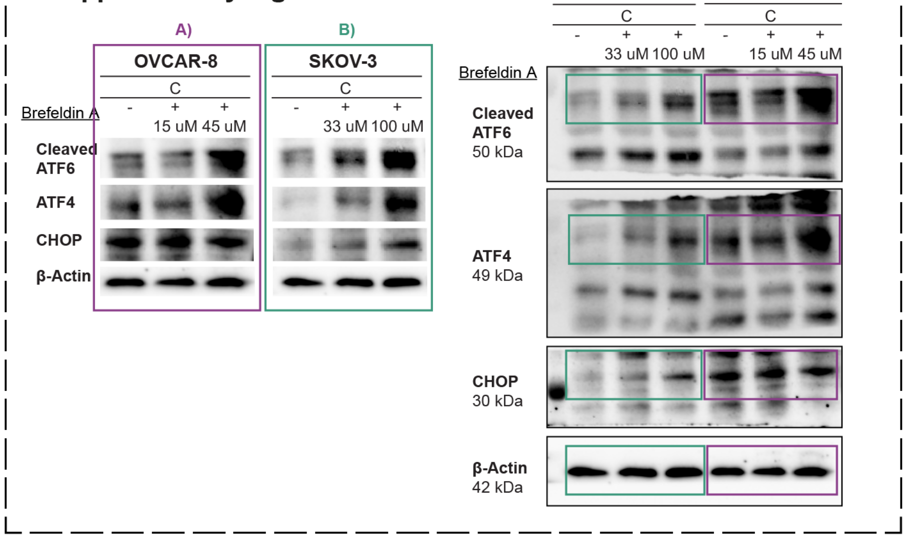

Supplementary Figure 5B

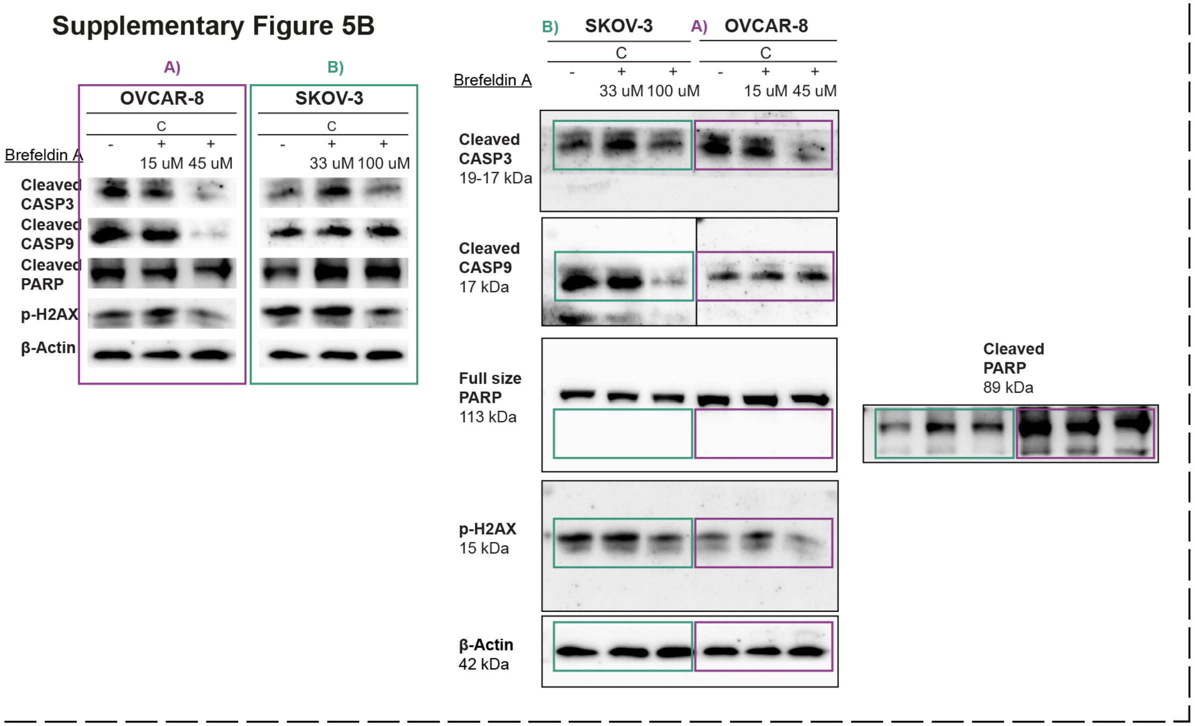

SUPPLEMENTARY FIGURE 1C

Detector	Sample	Avg delta Ct	Delta delta CT	Control Mean	delta Ct SD	Relative	Sample	Avg delta Ct	Delta delta CT	delta Ct SD	Relative	Sample	Avg delta Ct	Delta delta CT	delta Ct SD	Relative
Hook2	C1	13,505707	8,5976E-05	8,55035E-05	0,07757381	1,00552542	7(1)	13,882782	6,62013E-05	0,08769317	0,7742517	42(1)	13,688551	7,57418E-05	0,10450494	0,88583233
Hook2	C2	13,612358	7,98495E-05	8,55035E-05	0,12533852	0,93387318	7(2)	14,204053	5,29851E-05	0,05705086	0,61968354	42(2)	13,988697	6,15152E-05	0,09231453	0,71944662
Hook2	C3	13,428774	9,06852E-05	8,55035E-05	0,1056522	1,0606014	7(3)	13,494932	8,66205E-05	0,11555345	1,01306346	42(3)	13,88274	6,62032E-05	0,11440271	0,77427424

Detector	Sample	Avg delta Ct	Delta delta CT	Control Mean	delta Ct SD	Relative	Sample	Avg delta Ct	Delta delta CT	delta Ct SD	Relative	Sample	Avg delta Ct	Delta delta CT	delta Ct SD	Relative
Hook2	Scr 1	8,402691	0,002954867	0,005791299	0,07757381	0,51022529	shA 1	7,0135784	0,007739315	0,04612494	1,33636947	shD 1	8,649479	0,002490275	0,06511959	0,43000291
Hook2	Scr 2	7,5640793	0,005284273	0,005791299	0,12533852	0,91245039	shA 2	7,2843685	0,006414851	0,08257156	1,10767053	shD 2	8,009838	0,003879703	0,05648558	0,66991935
Hook2	Scr 3	6,774418	0,009134757	0,005791299	0,1056522	1,57732432	shA 3	7,023266	0,00768752	0,0948821	1,3274259	shD 3	7,6920166	0,004835844	0,04213814	0,83501888

	Relative		
	Control	CR2.7	CR2.42
	1,00552542	0,7742517	0,885832328
	0,93387318	0,61968354	0,71944662
Hook2	1,0606014	1,01306346	0,774274242

	Relative		
	Scr	shA	shD
	0,51022529	1,33636947	0,43000291
	0,91245039	1,10767053	0,66991935
Hook2	1,57732432	1,3274259	0,83501888
